# Pathogenic germline variants identified in pNEN patients during genetic testing

**DOI:** 10.1530/EO-25-0088

**Published:** 2026-05-28

**Authors:** Tara Arouchian, Alexandra Gangi, Ka Wing Fung, Teodora Dumitra, Abrahm Levi, Arsen Osipov, Andrew E Hendifar

**Affiliations:** ^1^ Samuel Oschin Comprehensive Cancer Center, Cedars Sinai Medical Center, Los Angeles, California, USA; ^2^ Department of Surgery, Cedars-Sinai Medical Center, Los Angeles, California, USA

**Keywords:** pancreatic neuroendocrine neoplasms, germline variants, DNA repair genes, precision medicine

## Abstract

Poor outcomes in pancreatic neuroendocrine neoplasms (pNENs) partially result from the inability to identify early-stage disease in patients. Prevention strategies have focused on identifying high-risk patients through genetic susceptibility genes. This study aims to report the incidence of pathogenic germline variants (PGVs) in pNEN. 129 pNEN patients that underwent germline testing with Invitae between September 5, 2019, and February 15, 2022, were retrospectively analyzed. PGVs were found in 14.7% (19/129) of pNENs. PGVs in pancreatitis genes were detected in 7.7% (3/39) of the 39 pNEN patients with an available pancreatitis panel. *CFTR* alterations were the most common pancreatitis-associated gene identified (5.1%, 2/39). *MUTYH* alterations were the most frequently detected (3.9%, 5/129) PGV in pNENs. DNA or base repair PGVs were found in 7.8% (10/129). Our findings suggest that germline testing may play a role in the standard-of-care management of pNEN. *MUTYH* alterations merit further evaluation as a potential risk factor for pNEN.

## Introduction

Pancreatic neuroendocrine neoplasms (pNENs) are a heterogeneous group of tumors originating from the neuroendocrine cells of the pancreas, encompassing well‐differentiated pancreatic neuroendocrine tumors (pNETs) and poorly differentiated pancreatic neuroendocrine carcinomas (pNECs) (1). The incidence of pNEN in the United States has increased over the years (2, 3), reaching 1.5 per 100,000 population in 2017 from 0.2 per 100,000 population in 1976, accounting for approximately 15% of all neuroendocrine neoplasms (3). Among neuroendocrine neoplasms of different primary tumor sites, pNEN has the worst prognosis, characterized by the highest proportion of distant-stage disease at diagnosis (40.3%) and highest mortality risk (4). The 5-year overall survival of metastatic pNEN was only 28% (5). The poor outcomes are in part due to the inability to identify patients with early-stage disease. As a result, prevention strategies have been directed toward the identification of patients at elevated risk through genetic screening.

Germline alterations account for approximately 10% of pNENs, which commonly occur as a manifestation of hereditary syndromes such as multiple endocrine neoplasia type 1 (MEN1), multiple endocrine neoplasia type 4 (MEN4), von Hippel–Lindau disease (VHL), neurofibromatosis type 1 (NF1), and tuberous sclerosis complex (6). Prior studies have identified several pathogenic germline variants (PGVs) in pNEN, which include, but are not limited to, *MEN1*, *VHL*, *MSH2*, and DNA repair genes, such as *BRCA1*, *BRCA2*, *CHEK2*, and *ATM* (7, 8, 9, 10, 11). However, the reported prevalence of these germline alterations varies widely across studies. Studies show that chronic pancreatitis predisposes patients to pancreatic malignancies, but limited data exist on the role of chronic pancreatitis-associated germline mutations in pNEN oncogenesis (12). This study aims to report and evaluate the incidence of germline alterations in pNEN and contribute to a more comprehensive genetic profile. Understanding the germline variant landscape of pNEN is crucial for improving risk assessment and guiding personalized management approaches.

## Methods

Invitae, a genetic testing company, initiated the Detect Hereditary Pancreatic Cancer Program (DETECT), which offered no-charge genetic testing for all individuals diagnosed with pNEN or pancreatic ductal adenocarcinoma (PDAC) seen at Cedars-Sinai. The program was launched on September 5, 2019, and closed on February 15, 2022. Participants underwent germline testing with either the Invitae Common Hereditary Cancers Panel (42–47 genes) or Multi-Cancer Panel (80–84 genes) with the option to add genes associated with chronic pancreatitis (*CFTR*, *CASR*, *CTRC*, *CPA1*, *PRSS1*, and *SPINK1*). Clinical data, including age, gender, ancestry, family history, and cancer stage, were collected from institutional medical records retrospectively. Only data made available by patients in these categories were analyzed. The incidence of PGVs in pNEN patients enrolled in this program was evaluated. This study was conducted retrospectively in compliance with the Declaration of Helsinki and fully utilized de-identified data. Therefore, informed consent was not required.

## Results

In total, 129 pNEN patients who underwent germline testing were retrospectively analyzed. The median age at testing was 58.0 years, and 47.3% (61/129) were female. Of the patients with available ancestry (*n* = 118), most patients were white (89/118, 75.4%). Ten patients (8.5%) were Jewish, 6 (5.1%) were Black/African American, 5 (4.2%) were Asian, 4 (3.4%) were Hispanic, and 4 (3.4%) identified as others. Of the patients with available family history (*n* = 48), cancer was reported in 81.3% (39/48) of patients. Of the patients with available cancer staging (*n* = 36), 52.8% (19/36) of patients had metastatic disease (Table 1).

**Table 1:** Demographic data of patients with pancreatic neuroendocrine neoplasm (pNEN).

	pNEN (*n* = 129)
Age at testing	
Median	58.0
Gender	
Female	61 (47.3%)
Male	68 (52.7%)
Ancestry	
Missing	11
Asian	5 (4.2%)
Black/African-American	6 (5.1%)
Hispanic	4 (3.4%)
Jewish	10 (8.5%)
White	89 (75.4%)
Others	4 (3.4%)
Family history	
Missing	81
No	9 (18.8%)
Yes	39 (81.3%)
Cancer stage	
Missing	93
0/1A/1B	9 (25.0%)
IIA/IIB	6 (16.7%)
III	2 (5.6%)
IV	19 (52.8%)

PGVs were found in 14.7% (19/129) of patients with pNENs (Table 2). One patient (1/129) had more than one PGV. Alterations in *MUTYH*, associated with polyposis syndrome, were the most frequently detected PGV in pNENs (5/129). DNA or base repair PGVs, including *ATM* (1/129), *CHEK2* (1/129), *FANCA* (1/129), *MUTYH* (5/129), *RAD50* (1/129), and *NTHL* (1/129), were found in 7.8% (10/129) of pNENs. Germline alterations were found in other genes, including *CDKN2A* (1/129), *HOXB13* (1/129), *MEN1* (2/129), *MITF* (1/129), *RET* (1/129), and *VHL* (1/129). Germline alterations in pancreatitis genes, *CFTR* (2/39) and *PRSS1* (1/39), occurred in 7.7% (3/39) of patients with a pancreatitis panel, with *CFTR* alterations as the most common pancreatitis-associated PGV in pNENs.

**Table 2:** Incidence of pathogenic germline variants (PGVs) of interest in pancreatic neuroendocrine neoplasm (pNEN). All PGVs identified are monoallelic.

Variable	pNEN
Patients with PGVs (*n* = 129)	19 (14.7%)
No. of mutated genes	
1	18 (14.0%)
2	1 (0.8%)
Pancreatitis gene PGVs (*n* = 39)	3 (7.7%)
CFTR	2 (5.1%)
PRSS1	1 (2.6%)
DNA/base repair gene PGVs (*n* = 129)	10 (7.8%)
MUTYH	5 (3.9%)
ATM	1 (0.8%)
CHEK2	1 (0.8%)
FANCA	1 (0.8%)
NTHL1	1 (0.8%)
RAD50	1 (0.8%)
Other PGVs (*n* = 129)	7 (5.4%)
MEN1	2 (1.6%)
CDKN2A	1 (0.8%)
HOXB13	1 (0.8%)
MITF	1 (0.8%)
RET	1 (0.8%)
VHL	1 (0.8%)

## Discussion

Our study revealed a PGV prevalence of 14.7%, which falls within the broad range of 7.5–33%, aligning with the overall trend reported in existing studies (7, 8, 9, 10, 11) (Table 3). Differences in cohort composition, the methodology and scope of genetic testing performed, and the criteria applied for the variant classification likely contribute to the variability observed across studies.

**Table 3:** Comparison of pathogenic germline variants (PGVs) identified in pancreatic neuroendocrine neoplasm (pNEN) studies.

Study (year)	Cancer type^a^	Cohort size (pNEN only)	% of patients with PGVs^a^	Most frequently detected PGVs and DNA repair gene variants^b^	Testing method
This study (2025)	pNEN	129	14.7%	*CFTR* (*n* = 2, 5.1%^c^); DNA repair genes (*n* = 9, 7.0%) including *MUTYH* (*n* = 5, 3.9%), *ATM* (*n* = 1, 0.8%), *CHEK2* (*n* = 1, 0.8%), *RAD50* (*n* = 1, 0.8%), and *FANCA* (*n* = 1, 0.8%)	Multi-gene panel (42–84 genes) with optional chronic pancreatitis-associated genes
Mohindroo *et al.* (2024) (10)	pNET	132	33.3%	*MEN1* (*n* = 25, 18.9%); *VHL* (*n* = 5, 3.8%); *MSH2* (Lynch syndrome) (*n* = 3, 2.3%); *MUTYH* (*n* = 2, 1.5%)^d^; and DNA repair genes (*n* = 8, 6.1%) including *BRCA1* (*n* = 3, 2.3%), *BRCA2* (*n* = 3, 2.3%), *BRIP* (*n* = 1, 0.8%), and *BARD* (*n* = 1, 0.8%)	Multi-gene panel (83–86 genes), targeted testing, physician reports
High-risk cohort					
Mohindroo *et al.* (2024) (10)	pNET	106	20.8%	DNA repair genes (*n* = 9, 8.5%) including *PALB2* (*n* = 1, 0.9%), *RAD50* (*n* = 1, 0.9%), *NTHL1* (*n* = 1, 0.9%), *BLM* (*n* = 1, 0.9%), *TP53* (*n* = 1, 0.9%), *BRCA2* (*n* = 1, 0.9%), *RECQL2* (*n* = 1, 0.9%), *BRCA1* (*n* = 1, 0.9%), and *ATM* (*n* = 1, 0.9%)	Multi-gene panel (83–86 genes), targeted testing, physician reports, clinical diagnosis
Unselected cohort					
Venizelos *et al.* (2023) (9)	pNEN	40^e^	7.5%	*MYOC* (*n* = 1, 2.5%); DNA repair genes (*n* = 2, 5%) including *MUTYH* (*n* = 1, 2.5%) and *FANCA* (*n* = 1, 2.5%)	Targeted sequencing of 360 cancer-related genes
Yachida *et al.* (2022) (7)	pNEN	66^f^	9.1%	*VHL* (*n* = 2, 3.0%); DNA repair gene including *PALB2* (*n* = 1, 1.5%) and *FANCG* (*n* = 1, 1.5%)^g^	Whole-genome sequencing (WGS) and whole-exome sequencing (WES)
Raj *et al.* (2018) (11)	pNET	88	15.9%	*APC* (*n* = 3, 3.4%); DNA repair genes (*n* = 9, 7.0%) including *MUTYH* (*n* = 4, 4.5%) and *CHEK2* (*n* = 4, 4.5%)	Targeted sequencing of 76 cancer susceptibility genes
Scarpa *et al.* (2017) (8)	pNET	102	16.7%	Specifically, mutations were found in *MEN1*, *VHL*, and DNA repair genes including *MUTYH*, *CHEK2*, and *BRCA2*, but the study did not provide detailed individual mutation frequency	Whole-genome sequencing (WGS)

^a^ pNEN, pancreatic neuroendocrine neoplasm; pNET, pancreatic neuroendocrine tumor.

^b^ Percentages were calculated or recalculated based on reported number of cases and total cohort size for consistency across studies.

^c^ Tested in a subset of 39 patients with genes associated with chronic pancreatitis.

^d^ *MUTYH* is considered one of the DNA repair genes in our study but was not included in the DNA repair grouping in the original publication.

^e^ Study cohort includes high-grade gastroenteropancreatic neuroendocrine neoplasms; only pNEN cases (*n* = 40) are included here.

^f^ Study cohort includes neuroendocrine neoplasms of the gastrointestinal system; only pNEN cases (*n* = 66) are included here.

^g^ Genes reported were derived from supplementary data of Yachida *et al.* (7). *MAD1L1*, while listed in the main text being identified in a pNEN patient, was identified in a duodenal neuroendocrine carcinoma patient per supplementary data. Only variants detected in pNEN patients are included here.

*MUTYH*, which was also detected in other cohorts (8, 9, 10, 11), was the most frequently detected altered gene in our study (*n* = 5, 3.9%). Although not statistically significant, the relatively high frequency of *MUTYH* compared with other genes in the cohort may suggest potential relevance in pNEN oncogenesis and merits further evaluation. One study reported germline mutations in 17 of 23 patients with MUTYH alterations and found associations between aggressive, high-grade disease and MUTYH-associated metastatic pNETs (13). Additionally, an association has been suggested by a recent report of ten patients with *MUTYH*-associated polyposis due to biallelic *MUTYH* PGVs, where all ten individuals had colorectal polyposis, five had colorectal cancer, and one developed a pNEN (14). Although biallelic loss of *MUTYH* is associated with colorectal polyposis and risk of colorectal cancer, this study suggests further evaluation into monoallelic pathogenic *MUTYH* alterations as a potential risk factor for pNEN as well.

Previous studies suggest that patients with chronic pancreatitis bearing alterations in *CFTR, SPINK1,* and *PRSS1* have an increased risk of developing pancreatic malignancies (15). Less is known about pancreatitis genes and their prevalence in pNENs specifically. *CFTR*, the most common pancreatitis-associated gene, was observed in two individuals (5.1%, 2/39) who underwent pancreatitis panel germline testing in our cohort, although it has not been widely documented in prior studies. However, the limited number of observations has restricted our ability to perform meaningful statistical analyses. Additionally, DNA repair PGVs, including *MUTYH*, *ATM*, *CHEK2*, *RAD50, NTHL1,* and *FANCA*, were identified in 7.8% (10/129) of our cohort. While these variants have been reported in other studies, the overall contribution of DNA repair genes to pNEN development remains uncertain as most were detected at a low frequency. These findings, taken together, demonstrate the necessity of further investigation in larger populations.

In addition to DNA/base repair and pancreatitis-associated PGVs, additional germline variants were identified in this cohort of pNENs, including *MEN1, VHL, CDKN2A,* and *MITF. MEN1* and *VHL* alterations are widely associated with pancreatic neuroendocrine tumors, and their presence in our cohort is consistent with previous studies (16, 17). *CDKN2A* alterations, as well as mutations in other tumor suppressor genes, are associated with pancreatic malignancies. A recent study highlighted the potential role of CDKN2A copy number losses in metastatic pNENs (18). Whereas the study is limited to homozygous deletions, our findings indicate that monoallelic *CDKN2A* alterations may also be relevant in pNENs. Additionally, one patient in our cohort presented with a *MITF* alteration. A 2024 study revealed that MITF-mediated epithelial–mesenchymal transition plays a role in pNEN progression, suggesting MITF as a relevant target in tumor development (19). Future studies in larger cohorts are needed to validate these findings.

Of the 19 patients with PGVs in our cohort, one patient had a HOXB13(G84E) mutation. The *HOXB13* gene has recently gained attention as a prominent predictive marker in prostate cancer. Individuals with germline *HOXB13* alterations, specifically G84E mutations, have a significantly higher incidence of prostate cancer than individuals without the mutation (20). Similar to their role in prostate development, HOX genes are also involved in the early development of the pancreas (21). With limited research surrounding the prevalence of HOX gene mutations in pancreatic cancer, it is unclear how specific HOX gene alterations may contribute to the development of pancreatic malignancies. Together with prior research, our findings identify *HOXB13* as a gene of interest warranting further investigation.

A *RET* alteration was present in one patient from our cohort. Alterations in the *RET* proto-oncogene are directly related to neuroendocrine syndromes, including multiple neoplasia type 2 (MEN2) (22). While *RET* alterations are more commonly associated with neuroendocrine tumors of neuronal crest cells, such as medullary thyroid carcinomas (MTCs) and pheochromocytomas, their role in pNEN oncogenesis is understudied (22). *RET* alterations are targetable using tyrosine-kinase inhibitors (23). Additionally, a recent phase 1 trial demonstrated the preliminary efficacy of BOS172738, a selective RET inhibitor, in *RET*-mutant MTC (24). Understanding the prevalence of *RET* mutations in pNENs may broaden treatment options for patients. Future studies should aim to identify PGVs in a larger pNEN cohort to create a more comprehensive genetic profile in this patient population.

In conclusion, our findings of PGVs in pNEN were consistent with previous studies suggesting the use of germline testing in the standard-of-care management of these patients while contributing to a broader understanding of the germline variant landscape in pNEN. The relatively high frequency of *MUTYH* alterations compared with other genes in this study may suggest their potential relevance in pNEN development. These findings provide new insights into the genetic heterogeneity of pNEN and highlight the significance of further research in larger populations to clarify the role of specific germline alterations in pNEN oncogenesis.

## Declaration of interest

The authors declare that there is no conflict of interest that could be perceived as prejudicing the impartiality of the work reported.

## Funding

This work did not receive any specific grant from any funding agency in the public, commercial, or not-for-profit sector.
